# Assessing attitudes towards medical assisted dying in Canadian family medicine residents: a cross-sectional study

**DOI:** 10.1186/s12910-019-0440-4

**Published:** 2019-12-27

**Authors:** Aaron Wong, Amy T. Hsu, Peter Tanuseputro

**Affiliations:** 1 Department of Family Medicine, University of Ottawa, Ottawa, Ontario Canada; 2 Clinical Epidemiology Program, Ottawa Hospital Research Institute, Ottawa, Ontario Canada; 3 Bruyère Research Institute, Ottawa, Ontario Canada; 4 Department of Medicine, University of Ottawa, Ottawa, Ontario Canada

**Keywords:** Medical assistance in dying, residents, Medical education, assisted suicide, Physician hastened death, Euthanasia, End-of-life, palliative care

## Abstract

**Background:**

Medical Assistance in Dying (MAID) in Canada came into effect in 2016 with the passing of Bill C-14. As patient interest and requests for MAID continue to evolve in Canada, it is important to understand the attitudes of future providers and the factors that may influence their participation. Attitudes towards physician hastened death (PHD) in general and the specific provision of MAID (e.g., causing death by lethal prescription or injection) are unknown among Canadian residents. This study examined residents’ attitudes towards PHD and MAID, and identified factors (e.g., demographics, clinical exposure to death and dying) that may influence their decision to participate in PHD and provide MAID.

**Methods:**

A cross-sectional survey was adapted from prior established surveys on MAID to reflect the Canadian setting. All Canadian family medicine programs were invited to participate. The survey was distributed between December 2016 and April 2017. Analysis of the results included descriptive statistics to characterize the survey participants and multivariable logistic regressions to identify factors that may influence residents’ attitudes towards PHD and MAID.

**Results:**

Overall, 247 residents from 6 family medicine training programs in Canada participated (response rate of 27%). While residents were most willing to participate in treatment withdrawal (52%), active participation in PHD (41%) and MAID by prescription of a lethal drug (31%) and lethal injection (24%) were less acceptable. Logistic regressions identified religion as a consistent and significant factor impacting residents’ willingness to participate in PHD and MAID. Residents who were not strictly practicing a religion were more likely to be willing to participate in PHD (OR = 17.38, *p* < 0.001) and MAID (lethal drug OR = 10.55, *p* < 0.01, lethal injection OR = 8.54, *p* < 0.05). Increased clinical exposure to death and dying crudely correlated with increased willingness to participate in PHD and MAID, but when examined in multivariable models, only a few activities (e.g., declaring death, completing a death certificate) had a statistically significant association. Other significant factors included the residents’ sex and location of training.

**Conclusions:**

Residents are hesitant to provide MAID themselves, with religious faith being a major factor impacting their decision.

## Background

The legalization of Medical Assistance in Dying (MAID) in Canada came into effect in 2016 with the passing of Bill C-14. MAID was defined in Bill C-14 as (a) the administering or prescribing by a clinician of a substance to a person, at their request, that causes their death; or (b) the prescribing or providing by a clinician of a substance to a person, at their request, so that they may self-administer the substance and in doing so cause their own death [1]. Prior to this legislation, MAID was prohibited by the Criminal Code of Canada which stated that anyone who aids or abets a person in committing suicide would commit an indictable offence and that no person may consent to death being inflicted on them [2]. As a consequence of this prohibition and prior to the passing of Bill C-14, consenting patients with a grievous and irremediable medical condition (e.g., end stage amyotrophic lateral sclerosis) who experienced enduring intolerable suffering were only offered palliative care.

A recent Canadian survey of 1407 physicians showed that only 29% would consider providing MAID if it was requested by a patient [3]. Given the implications of Bill C-14 on the future practice of medicine, it is also important to understand medical residents’ attitudes towards MAID and the factors that contribute to their decision to participate. All residents will, at one time during their training, find themselves working in tertiary care centres, providing care to hospitalized patients who are frail and may be suffering from a grievous and irremediable medical condition (i.e., patients who would be eligible for MAID under Bill C-14). Yet, studies in the U.S. and Canada indicate that residents may not be receiving adequate training in providing end-of-life care, nor are they taught the medical ethics of providing care to a potentially non-autonomous, dying patient [4–6]. This gap in medical training will be further complicated by the introduction of MAID, as well as the lack of experience among educators to teach residents how to field requests for assisted dying.

Results from existing U.S.-based studies suggest that residents are hesitant to participate in MAID (1–40%), but may be supportive of their colleagues’ involvement [7–11]. Similarly, in Mexico, residents have expressed limited support (18–29%) for MAID [12, 13]. These studies, compared to the results from surveys of practicing physicians, indicate that residents may be more willing to participate in MAID than established physicians [7, 9, 10, 13, 14]. These studies also suggest that religion, gender, and the amount of years in clinical practice may also impact medical residents’ decision to participate in MAID.

Until recently, however, there have not been any formal investigations of Canadian residents’ perceptions of or willingness to participate in MAID. MacDonald et al.’s study [6] of Canadian family medicine residents’ attitudes towards MAID was the first of its kind. From their survey of 71 preceptors and 62 residents in the Queen’s University family medicine residency program, they found that most residents would participate in MAID (69%). However, the generalizability of their findings is limited by the single-site study design. Furthermore, while they found that residents may be willing to participate passively as an observer or as a part of a team providing MAID, it’s unclear if they would actively provide MAID on their own. This distinction is important because active participation raises stronger ethical concerns for clinicians. Studies have demonstrated that physicians are accepting of indirect end-of-life activities such as withholding or withdrawing life sustaining treatments (i.e., an active decision-making process to stop or not start a given intervention that would prevent a patient from dying), but not physician-assisted suicide [15].

While MAID, in a strict sense, refers to the administration or provision of a substance that ends the patients’ life, addressing a patient’s request for MAID in clinical practice is more complex. It requires exploring the nature of the patient’s request, facilitating their request, and assessing their eligibility. In our study, we used the term “participate in physician hastened death (PHD)” to refer to participation in MAID in a broad sense. This would include exploring patients’ requests and assessing eligibility in addition to the provision of MAID by lethal prescription or injection. In this respect, MAID can be seen as a part of a continuum of end-of-life care that ranges from discontinuing or withholding treatment, to actively participating in PHD, and finally the provision of MAID.

As the opinions and factors that shape Canadian residents’ willingness to participate in MAID have not been fully described, the objectives of our study were to: [1] Describe Canadian family medicine residents’ attitudes towards PHD and the provision of MAID; and, [2] identify the factors (e.g., demographics, clinical exposure to death and dying) that may influence their decision to actively provide MAID.

## Methods

### The survey

We conducted a cross-sectional study of family medicine residents in Canada to determine their opinions on PHD and MAID and the factors that may influence their decision to participate. All family medicine programs in Canada were invited to participate. We included residents in their postgraduate year (PGY) 1 or PGY2 but excluded medical students and staff physicians. Residents in enhanced skills programs (PGY3) were also excluded as some of them could be pursuing specialization in palliative care. As there were no previous Canadian studies at the time to base a survey instrument on, we adapted our survey from studies conducted in the U. S and Mexico [5, 11, 12, 14]. We modified some questions to reflect the Canadian context (e.g., legal definitions, eligibility) while preserving questions that have shared meanings and were found to be prior predictors of participation in MAID (e.g., religion, gender, years in practice). The survey was pilot tested, internally, for face validity by three staff physicians from the Department of Palliative Care at The Ottawa Hospital. This study was approved by the Ottawa Health Science Network Research Ethics Board.

An e-mail containing the survey link, consent form, and recruitment letter was sent to participants by their program coordinators. Participation was voluntary and anonymous. The survey was made available between December 2016 and April 2017 in English or French. In the end, 6 of 17 programs were included, which represented a total of 839 eligible participants. Programs that were excluded from this study either did not respond to our invitation or grant permission to distribute our survey.

### Metrics

Our survey (See Additional file 1: Questionnaire on MAID) captured residents’ demographic characteristics (age, gender, ethnicity, training, and faith), clinical exposure to death and dying (e.g., managing pain and suffering, declaring death) and attitude towards PHD and MAID using an example of a patient who fulfills all the criteria for MAID under Bill C-14. Residents’ agreement with various statements (e.g., would you believe the patient is asking for PHD; assess for incapacity; withdraw treatment; participate in PHD; provide MAID by lethal prescription or injection) was captured using Likert scales that included an option for “unsure”.

### Analysis

Data analysis was performed in R version 3.4.0. Statistical significance was set at *p* < 0.05 and, where relevant, we also reported differences at *p* < 0.01 and *p* < 0.001. Participants with missing responses (*n* = 26) were not excluded from the study, but the missing values were excluded from the calculation of the descriptive statistics and the regression models. Due to the limited sample size, the Likert scale responses were subsequently collapsed into residents who would willingly participate (“agree” and “strongly agree”), those who would not participate (“disagree” and “strongly disagree”), and those who were neutral or unsure (“undecided” and “neutral”) for the analysis. Clinical exposure to death and dying was also summarized as categorical variables (e.g., 0, 1–10, and 11+ cases). Logistic regression models were used to examine the significance of descriptive variables on residents’ willingness to participate in PHD and MAID.

## Results

### Demographic characteristics

Overall, 6 of 17 family medicine residency programs participated, including the University of Saskatchewan, University of Manitoba, University of Toronto, University of Ottawa, McGill University and Laval University. These institutions represent 4 of the 10 provinces in Canada (Saskatchewan, Manitoba, Ontario and Quebec). For the analysis, we further collapsed them into three regions: Ontario (University of Toronto, University of Ottawa), Quebec (McGill University, Laval University) and Prairie (University of Saskatchewan, University of Manitoba). In total, 247 residents participated in the survey with an overall response rate of 27%. Their characteristics are described in Table 1. The mean age was 28.8 years (*SD* = 29.9 years). There were more female (*n* = 175) than male (*n* = 72) participants. Christians (48.6%) were the largest religious group, followed by those who were non-religious (34.4%) and of other religions (15.8%), which included participants who self-identified as Jewish, Muslim, Buddhist, Hindu, Sikh, Aboriginal, or all others. The largest ethnic group in our study was Caucasians (73.7%).

**Table 1:** Proportion of Residents Answering “Agree” by Demographic

Variable	Category	TOTAL	Withdraw Treatment		Participate in PHD		Prescribe Lethal Drug		Administer Lethal Injection	
			n	(% total)	n	(% total)	n	(% total)	n	(% total)
Age (yrs)	(mean)	28.8	29.9	–	29.1	–	29.1	–	29.3	–
	(SD)	4.5	3.8	–	4.0	–	4.1	–	4.4	–
Sex	Male	72	34	(55.7)	28	(45.9)	24	(40.7)	17	(27.9)
	Female	175	76	(51.0)	59	(39.3)	41	(27.3)	34	(22.7)
School	Quebec	91	29	(34.9)***	36	(42.9)	29	(34.9)	26	(31.0)
	Ontario	92	52	(64.2)	36	(44.4)	26	(32.1)	18	(22.2)
	Prairie	53	29	(63.0)	15	(32.6)	10	(22.2)	7	(15.2)
PGY	1	139	61	(49.6)	52	(41.9)	42	(33.9)	35	(28.2)
	2	106	49	(57.6)	33	(38.8)	23	(27.7)	16	(18.8)
Religion	Not religious	85	38	(50.0)	41	(53.9) ***	26	(34.7)	24	(31.6) *
	Other	39	22	(68.8)*	16	(50.0) *	13	(41.9)	9	(28.1)
	Christian	120	48	(48.5)	29	(29.0)	25	(25.0)	17	(17.0)
Practice	Strictly	40	18	(60.0)	3	(10.0)	2	(6.7)	1	(3.3)
	Not strictly	66	33	(61.1)	17	(30.9) *	15	(27.8) *	12	(21.8)
	Not	120	49	(45.4)	53	(49.1) ***	37	(34.6) **	29	(26.9) *
Ethnicity	Other	65	36	(65.5)	19	(34.5)	17	(30.9)	12	(21.8)
	Caucasian	182	74	(47.7)*	68	(43.6)	48	(31.2)	39	(25.0)
TOTAL		247	115	(52.0)	91	(40.9)	68	(30.9)	54	(24.3)

Proportion of participants that answered “agree” to different end-of-life activities.

(% total) - % of participants responding “agree” by survey question (column) within each demographic (row)

PHD - Physician Hastened Death. PGY – Post graduate year.

Statistical significance calculated as described in methods with *** *p* value < 0.001, ** *p* value < 0.01, * *p* value < 0.05.

### Willingness to participate in PHD and MAID

The proportion of residents who agreed with different activities across the spectrum of end-of-life care is summarized in Table 1 and is organized in the order of increasing involvement (i.e., from treatment withdrawal to administering a lethal injection). Willingness to participate decreased with more direct forms of end-of-life care. For example, while 52.0% of residents were willing to withdraw treatment for a patient meeting eligibility criteria, fewer residents were willing to actively participate in PHD (40.9%), or participate in MAID by lethal prescription (30.9%) and lethal injection (24.3%).

The willingness to withdraw treatment varied across demographics. Quebec residents and Caucasian residents were significantly less willing to withdraw treatment. Male residents were more willing to withdraw treatment, although this difference from female residents was not statistically significant. Medical residents in the ‘other religions’ category were more willing to withdraw treatment than their counterparts. The willingness to participate in PHD differed across religions and the level of adherence to religious practices. Non-religious residents (53.9%) and those from other religions (50.0%) were more willing to participate in PHD compared to Christians (29.0%). Similarly, residents who were “not” or “not strictly” practicing their faith were more willing to participate in PHD than those with strict adherence to religious practices. The willingness to participate in MAID by lethal prescription was also notably higher among residents who were “not” (34.6%) or “not strictly” (27.8%) practicing their religion compared to those with strict adherence to religious practices (6.7%). Similarly, the proportion of residents who were willing to participate in MAID by lethal injection was higher among those who were not religious (31.6% vs. 17.0% for Christians) or “not practicing” their religion (26.9% vs. 3.3% for residents with strict adherence to their religious practices).

### Clinical exposure to death and dying

**Fig. 1:**
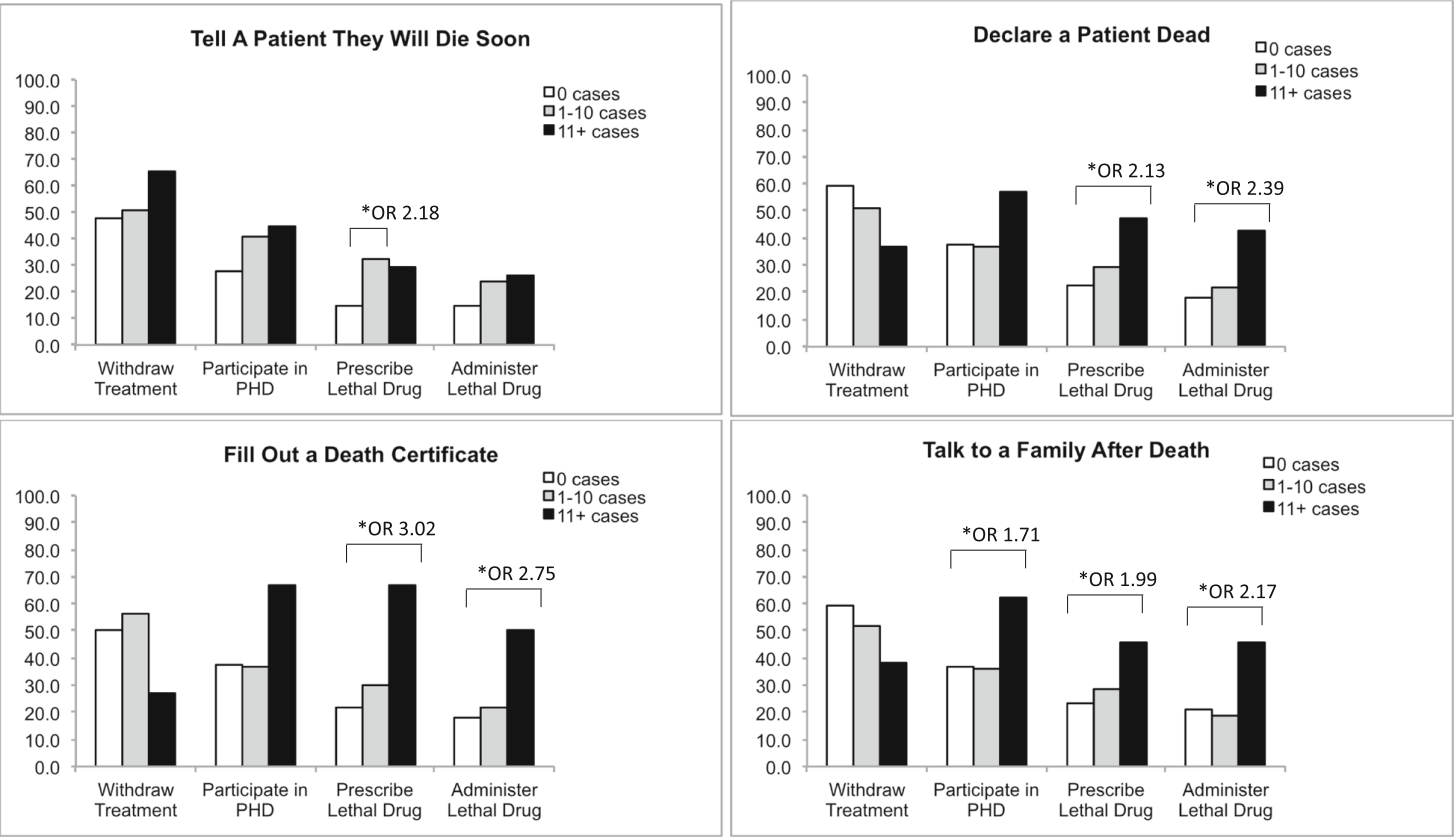
Proportion of Residents Answering “Agree” by Exposure to Death and Dying Figure 1 shows the proportion of participants that answered “agree” to different end-of-life activities among those with different levels of exposure to death and dying (0 cases vs. 1–10 cases vs. 11+ cases) and different exposures to death and dying. PHD - Physician Hastened Death**.** Statistical significance calculated as described in methods with *** *p* value < 0.001, ** *p* value < 0.01, * *p* value < 0.05′

Increased exposure to death and dying was correlated with greater odds of agreeing to participate in different end-of-life activities (Fig. 1). Experienced residents with 11+ cases of declaring death, completing death certificates, and talking to families after death were more likely to participate in MAID by lethal prescription or by lethal injection than residents with less experience. Experienced residents also displayed a trend towards greater willingness to participate in PHD overall. However, this correlation was only statistically significant among residents with exposure to talking to families after death.

### Predictors of resident participation in PHD and MAID

Logistic regressions were performed to determine which factors were significantly and independently correlated with different end-of-life activities (Table 2). Increased likelihood of willingness to withdraw treatment was seen in Ontario and Prairie residents compared to Quebec. Higher likelihood of willingness to participate in PHD was observed among residents “not” (OR = 17.38) or “not strictly” (OR = 5.17) practicing their religion, as well as residents practicing a non-Christian religion (OR = 3.84). The likelihood of willingness to participate in MAID by lethal prescription was increased if they were “not” practicing their religion (OR = 10.55) or practicing a non-Christian religion (OR = 3.57), but decreased if they were female (OR = 0.38). Similarly, higher likelihood of willingness to participate in MAID by lethal injection was seen among residents “not” practicing their religion (OR = 8.54).

**Table 2:** Logistic Regression Model to Estimate Probability of Residents “Agreeing” with End-of-Life Activities

Variable	Category	Withdraw Treatment (*n* = 188)		Participate in PHD (*n* = 189)		Prescribe Lethal Drug (*n* = 187)		Administer Lethal Injection (*n* = 189)	
		OR	95% CI	OR	95% CI	OR	95% CI	OR	95% CI
Age (in years)		1.0	0.93–1.07	1.05	0.97–1.13	1.02	0.95–1.10	1.05	0.97–1.14
PGY	1								
	2	0.81	0.41–1.59	0.72	0.35–1.45	0.74	0.34–1.57	0.67	0.29–1.50
Sex	Male								
	Female	0.99	0.49–1.97	0.57	0.27–1.19	0.38*	0.18–0.81	0.57	0.26–1.28
School	Quebec								
	Ontario	3.80***	1.84–8.10	1.15	0.54–2.47	0.80	0.36–1.77	0.54	0.23–1.24
	Prairie	3.57**	1.49–8.88	0.73	0.27–1.89	0.45	0.14–1.26	0.38	0.11–1.13
Religion	Not Religious								
	Other	1.13	0.36–3.65	3.84*	1.20–12.96	3.57*	1.09–12.11	1.49	0.42–5.15
	Christian	0.58	0.25–1.33	1.06	0.47–2.39	1.42	0.60–3.40	0.66	0.26–1.65
Practice	Strictly								
	Not strictly	1.24	0.44–3.41	5.17*	1.24–35.63	4.80	1.13–33.44	6.45	1.10–123.38
	Not	0.68	0.24–1.85	17.38***	4.16–122.97	10.55**	2.5–74.40	8.54*	1.49–162.75
Ethnicity	Other								
	Caucasian	0.55	0.26–1.16	1.19	0.54–2.67	0.75	0.33–1.70	0.86	0.37–2.10

Increased OR refers to more agreement. PHD - Physician Hastened Death. PGY – Post Graduate Year. OR – Odds ratio. 95% CI – 95% Confidence Intervals. Statistical significance calculated as described in methods with *** *p* value < 0.001, ** *p* value < 0.01, * *p* value < 0.05.

A sensitivity analysis was conducted to examine the independent effect of different levels of clinical exposures to death and dying with residents’ perspective on end-of-life activities. Although increased clinical exposure to death and dying was crudely correlated with increased willingness to participate in MAID (Fig. 1), when these were examined in multivariable models, only a few activities were significantly correlated with our outcomes (see Additional file 2: Table S1). Specifically, residents who had 11+ cases of declaring death or completing a death certificate were more likely to be willing to participate in MAID by lethal prescription and lethal injection. Additionally, residents who had more clinical exposure to death and dying (11+ cases) in the form of talking to and counselling family members after death had greater odds of expressing a willingness to participate in PHD and MAID by lethal injection.

## Discussion

Our study examined residents’ attitudes towards PHD and MAID and sought to determine factors that were associated with their future willingness to participate. While 40.9% of the residents would actively participate in PHD, fewer residents would participate in the provision of MAID by lethal prescription or lethal injection. Christians and residents with higher levels of religiosity were consistently less likely to be willing to participate in MAID. We also found that residents with higher levels of exposure to death and dying were more likely to be willing to participate in MAID. However, after adjusting for the effects of other factors in our logistic regression models, only a few specific exposures to death and dying remained statistically significant. Overall, the strongest predictors of willingness to participate in MAID from our multivariable analyses were: [1] Not practicing a religion, and [2] participating in a religion other than Christianity. Interestingly, our model also showed that Ontario and Prairie residents were more likely to withdraw treatment than their counterparts in Quebec, and that females were less likely to prescribe a lethal drug than males.

Our findings showed that non-religious residents and those from other religions were more likely to participate in PHD when compared to those who self-identified as Christian. Christian residents may be less likely to participate in MAID due to religious arguments which suggest that suicide is as morally objectionable as murder. Furthermore, the act of carrying out a request for assisted suicide represents an injustice that cannot be excused [16]. Though there are other religions (e.g., Judaism, Sikhism) and sects within specific religions that also oppose assisted deaths, our sample size may not have been large enough to detect these perspectives. Nonetheless, our observation based on comparing the views of residents who self-identified as Christians and those whose belief lies in other faiths is consistent with several others studies, which also found lower agreement to participate in MAID among Christians [8, 11, 13–15] and echoes the current opinions about MAID among some Christian physicians in Canada [17]. Although one of our regression models suggested that residents who are Christians may be more likely to participate in MAID by lethal prescription, the relationship was not statistically significant and the direction of association reversed when we removed the level of adherence to one’s religion from the model (results not shown); this suggests that Christian residents’ willingness to participate in MAID is strongly influenced by their level of adherence to their faith. Results from our regression models also suggested that residents from other religions were more likely to participate in PHD and MAID by lethal prescription than non-religious residents. Although this finding is interesting, the generalizability of this observation may be limited as our study population was predominantly Christian or non-religious, and the category of “other religion” is a conglomerate of different faiths. As variations in residents’ agreeability with MAID between these other religions have not been well-described, it is difficult to draw clear conclusions from this and should be explored in future studies.

Female residents were less likely to be willing to participate in MAID by lethal prescription than their male colleagues. We also observed that females were also less willing to participate in PHD and participate in MAID by lethal injection, although results were not statistically significant in our regression model. While this is noteworthy, existing literature have been inconclusive on the role of female gender and the influence it has on medical residents’ and physicians’ perspective on PHD or MAID; previous studies have suggested that female residents are less accepting of PHD [18], but others show no difference [9, 11] or even the opposite trend [12].

Quebec residents were less likely to respond to a request for MAID by withdrawing treatment than their colleagues in Ontario and the Prairies, but more likely to agree with the provision of MAID, although these differences were not statistically significant in our regression models. This was not surprising since Quebec was the first province to legalize assisted death [19] and has completed more cases of MAID than any other province in Canada [20]. Quebec has also sought clarification on the court’s definition of “reasonably foreseeable” in the assisted dying framework to broaden its access to those currently denied MAID [21]. As Quebec continues to push the boundaries of MAID, it could be speculated that residents who are training in Quebec are becoming more familiar and less hesitant to address requests for MAID when compared to their peers in other provinces. However, our study finds that the majority of Quebec residents are still hesitant to provide MAID. Their reluctance may be underscored by the fact that lethal prescription in Quebec is currently not a regulated practice under Quebec’s landmark end-of-life care bill that enabled patients to choose MAID, known as Bill 52 or the Act Respecting End-of-Life Care, and by the College of Physicians of Quebec.

Having additional years in clinical practice is likely to play a role in residents’ willingness to participate in MAID. Our study suggests that residents are more agreeable to participate in PHD than physicians in Canada (40.9% vs. 29%) [3] but less agreeable than Canadian medical students (71%) [22]. This should be interpreted with some caution, however, as each study’s phrasing of questions on MAID differed slightly and this is known to influence participants’ agreeability with MAID [15]. Nonetheless, this difference in attitude across different stages in clinical practice echoes previous U.S., Mexican and Canadian studies, which found that staff physicians were more hesitant to participate in MAID [6, 7, 10, 18]. In addition, one study demonstrated that residents with more years in clinical practice (e.g., fellows in their 5th–8th year of oncology training) were more hesitant to participate in PHD than residents in their first 3 years of post-graduate training [13]. Some studies have speculated that early residents may be more willing to “throw in the towel” when severe health complications arise, due to their clinical inexperience or poor knowledge of palliative care [8, 10]. Residents may also be unduly influenced by the severity of cases they encounter, the grueling hours of residency, apathy towards patients and “burnout” [8]. Our results suggest that residents with more exposure to death and dying actually had increased agreement to participate in MAID. This finding seems to contradict the idea that increased palliative care knowledge would lead to a decrease in participation in MAID. Further work will be needed to determine if palliative care knowledge specifically shapes residents’ attitudes towards MAID.

Our study showed that family medicine residents in Canada may be hesitant to participate in PHD and the provision of MAID. Despite our small sample size, this observation is consistent with other studies on residents’ standpoints on MAID [7–14, 18]. There remains some variability between our study and others that are likely due to differences in the methodology used as well as other social, demographical, and geographical factors of the study populations [15]. Since most existing studies were single-centred, opinions often only reflect the local acceptance of assisted death. For example, residents in our study were less willing to participate in MAID than a recent Canadian study at one university [6]. This difference could be explained by the sample population (being single-centred vs. multi-centred) or, possibly, because their scenario for residents was more passive (i.e., observing or participation in MAID as a part of a team), which has been shown to be more acceptable to physicians. As MAID is now a part of medical practice in Canada, more studies on regional variability are needed to inform the future training of our medical residents.

## Limitations

Our study is limited by its sample size (*n* = 247) and response rate (27%). Although our survey invited all training programs in Canada, we were unable to recruit residents from Western Canada and the Maritimes. Poor response rates are not uncommon for national surveys of residents in Canada; a recent national resident survey by the Resident Doctors of Canada had a response rate of 15.8% [23]. Nonetheless, our sample size is the largest among similar studies in North America [7–13, 18] and our overall findings are consistent with these studies. We were able to characterize Canadian residents’ attitudes towards MAID and address some concerns with past surveys, as ours was sent to multiple jurisdictions that operate under different provincial legislation, included multiple languages (to include French speaking residents), and used a simplified assisted dying case. Although the phrasing of our question regarding participation in PHD could have been more specific, it was intended to capture participation in MAID in a general sense, which would include indirect participation in MAID (exploration of patient ideas and assessment of eligibility) and the actual provision of MAID (lethal prescription and injection). Our study’s participants seemingly understood this difference implicitly, as suggested by the finding that residents were more agreeable to participation in PHD in general than the actual provision of MAID. Further studies focusing on this specific distinction would help clarify residents’ attitudes on indirect MAID activities. The inferences from our study may also be limited by the short data collection period (December 2016 to April 2017), which may have prevented greater participation and imply that our data may be reflective of residents’ perceptions only at that point in time. Our study examined the attitudes of family practice residents, which may not reflect of the attitudes of residents in other subspecialties. Finally, it would also have been interesting to assess other factors that may have influenced residents’ willingness to provide MAID such as their own psychological well-being, personal values, or views on the role of a doctor [24]. Future studies, where possible, could consider these factors as well as assess for possible changes in the perceptions of residents over time and between different specialties.

## Conclusion

As MAID in Canada evolves, residents will need to acquire the skills to provide end-of-life care that may include assisted death. Our study demonstrates that residents are mostly hesitant to provide MAID and that a major predictor of their willingness is religion. Sex and exposure to death and dying were also found to potentially affect the willingness of residents to provide MAID, although further study is needed to clarify these relationships.

## Supplementary information

**Additional file 1.** Questionnaire on MAID. Copy of the survey questions and the order that they were presented in.

**Additional file 2: Table S1.** Logistic Regression Examining Exposure to Death and Dying on Residents’ Willingness to Participate in MAID.

## Data Availability

The datasets used and/or analyzed during the current study are available from the corresponding author on reasonable request.

## Abbreviations

MAID: Medical Assistance in Dying
PGY: Post Graduate Year
PHD: Physician Hastened Death
REB: Research Ethics Board
