# Supplementary material for: Assessing attitudes towards medical assisted dying in Canadian family medicine residents: a cross-sectional study
Source: BMC Med Ethics. 2019 Dec 27;20:103. doi: 10.1186/s12910-019-0440-4 (PMC6935122; doi:10.1186/s12910-019-0440-4)
Supplement: Supplementary file 1 — Additional file 1. Questionnaire on MAID. Copy of the survey questions and the order that they were presented in. [file 12910_2019_440_MOESM1_ESM.docx]

**APPENDIX:**

**Questionnaire on MAID**

**-1- DEMOGRAPHICS**

What is your age # input

What is your gender M / F

Where are you doing your residency Memorial, Dalhousie
Laval, Sherbrooke, Montreal, McGill
Ottawa, Queens, Northern Ontario, Toronto, McMaster, Western
Manitoba, Saskatchewan
Alberta, Calgary

British Columbia

Other

What year of training are you in PGY 1 / 2

What religious/cultural background are you? (if any) Buddhist / Christian / Hindu / Jewish / Muslim / Sikh / Aboriginal / other / not religious

Are you currently practicing your religion strictly / not-strictly / no

What is your ethnicity Aboriginal / Asian / Black / Hispanic / Caucasian / other

**-2- EXPOSURE**

Since graduating medical school I have had to: estimate number of individual patients:

1. Manage pain/suffering at the end of life # input
2. Tell a patient they will die soon # input
3. Declare a patient dead # input
4. Fill out a death certificate # input
5. Talk to a family after death # input
6. Talk to a patient about assisted dying # input

Medical school has prepared me to provide palliative care S. Disagree / Disagree / Neutral / Agree / S. Agree / Unsure

Residency has prepared me to provide palliative care S. Disagree / Disagree / Neutral / Agree / S. Agree / Unsure

I am confident in my ability to provide palliative care S. Disagree / Disagree / Neutral / Agree / S. Agree / Unsure

**-3- PHYSICIAN HASTENED DEATH**

| The Supreme Court of Canada has recently legalized Physician Assisted Dying.  They have allowed Physician Assisted Dying for a competent adult person who:  (1) clearly consents to the termination of life,  (2) has a grievous and irremediable medical condition (including an illness, disease or disability), and  (3) experiences enduring suffering that is intolerable to the individual in the circumstances of his or her condition |
| --- |

**If your patient met the Supreme Court’s requirements for Physician Assisted Dying and asked you to “Help (him/her) die”, you would:**

| 1. Believe that the patient is asking for **Physician Hastened Death (PHD)** - *an intentional hastening of death performed with the assistance of a physician* | S. Disagree / Disagree / Neutral / Agree / S. Agree / Unsure |
| --- | --- |
| 2. Assess for incapacity, coercion, inconsistency, ambivalence, depression, social impairment or reversible causes of distress **BEFORE** considering PHD | S. Disagree / Disagree / Neutral / Agree / S. Agree / Unsure |
| 3. Agree that assessing (as in question 2) does **NOT** infringe upon patient autonomy | S. Disagree / Disagree / Neutral / Agree / S. Agree / Unsure |
| 4. Actively participate in PHD | S. Disagree / Disagree / Neutral / Agree / S. Agree / Unsure |
| 5. Administer a lethal injection | S. Disagree / Disagree / Neutral / Agree / S. Agree / Unsure |
| 6. Prescribe a lethal drug | S. Disagree / Disagree / Neutral / Agree / S. Agree / Unsure |
| 7. Address their request by discontinuing/withholding a treatment | S. Disagree / Disagree / Neutral / Agree / S. Agree / Unsure |
|  |  |
| 8. Choose to not actively participate but refer to a specific MD for PHD | S. Disagree / Disagree / Neutral / Agree / S. Agree / Unsure |
| 9. Not refer but provide information on how to access PHD services | S. Disagree / Disagree / Neutral / Agree / S. Agree / Unsure |

**Would you support a patient’s request for PHD (Physician Hastened Death) if they met the above criteria:**

| 10. and had a potentially reversible cause of distress and did not follow the suggested treatment? | S. Disagree / Disagree / Neutral / Agree / S. Agree / Unsure |
| --- | --- |
| 11. and have a prognosis of **years** | S. Disagree / Disagree / Neutral / Agree / S. Agree / Unsure |
| 12. and have a prognosis of **months** with **well controlled** physical/mental symptoms | S. Disagree / Disagree / Neutral / Agree / S. Agree / Unsure |
| 13. and have a prognosis of **months** with **poorly controlled** physical/mental symptoms | S. Disagree / Disagree / Neutral / Agree / S. Agree / Unsure |
