# Supplementary material for: Assessing attitudes towards medical assisted dying in Canadian family medicine residents: a cross-sectional study
Source: BMC Med Ethics. 2019 Dec 27;20:103. doi: 10.1186/s12910-019-0440-4 (PMC6935122; doi:10.1186/s12910-019-0440-4)
Supplement: Supplementary file 2 — Additional file 2: Table S1. Logistic Regression Examining Exposure to Death and Dying on Residents’ Willingness to Participate in MAID. [file 12910_2019_440_MOESM2_ESM.docx]

**SUPPLEMENTAL Table 1: Logistic Regression Examining Exposure to Death and Dying on Residents’ Willingness to Participate in MAID**

|  |  | **Participate in PHD (#5) (n=184)** | | | **Prescribe Lethal Drug (#3)**  **(n=183)** | | | **Prescribe Lethal Drug (#4)**  **(n=183)** | | |
| --- | --- | --- | --- | --- | --- | --- | --- | --- | --- | --- |
| Variable | Category | OR | 95% CI |  | OR | 95% CI |  | OR | 95% CI |  |
| Age (in years) |  | 1.04 | 0.97 - 1.13 |  | 1.01 | 0.93 - 1.09 |  | 1.03 | 0.95 - 1.12 |  |
| PGY | 1 | … | … |  | … | … |  | … | … |  |
|  | 2 | 0.73 | 0.33 - 1.60 |  | 0.56 | 0.24 - 1.28 |  | 0.43 | 0.17 - 1.03 |  |
| Sex | Male | … | … |  | … | … |  | … | … |  |
|  | Female | 0.44* | 0.20 - 0.95 |  | 0.37* | 0.17 - 0.81 |  | 0.39* | 0.18 - 0.86 |  |
| School | Quebec | … | … |  | … | … |  | … | … |  |
|  | Ontario | 1.13 | 0.58 - 3.09 |  | 1.21 | 0.50 - 2.94 |  | 0.97 | 0.41 - 2.25 |  |
|  | Prairie | 0.73 | 0.25 - 2.05 |  | 0.66 | 0.19 - 2.20 |  | 0.58 | 0.18 - 1.72 |  |
| Religion | Not Religious | … | … |  | … | … |  | … | … |  |
|  | Other | 3.41 | 0.99 - 12.30 |  | 3.89* | 1.12 - 14.22 |  | 3.29 | 0.93 - 12.14 |  |
|  | Christian | 1.06 | 0.46 - 2.46 |  | 1.58 | 0.64 - 3.97 |  | 1.44 | 0.57 - 3.69 |  |
| Practice | Strictly | … | … |  | … | … |  | … | … |  |
|  | Not strictly | 8.15* | 1.78 - 62.65 |  | 5.14 | 1.16 - 36.94 |  | 4.34 | 0.99 - 30.76 |  |
|  | Not | 21.09** | 4.67 - 160.56 |  | 11.13** | 2.57 - 79.55 |  | 7.96* | 1.79 - 57.88 |  |
| Ethnicity | Other | … | … |  | … | … |  | … | … |  |
|  | Caucasian | 1.36 | 0.59 - 3.26 |  | 0.61 | 0.26 - 1.44 |  | 0.64 | 0.27 - 1.51 |  |
|  |  |  |  |  |  |  |  |  |  |  |
| Exposure to Death and Dying: | |  |  |  |  |  |  |  |  |  |
| Declare a patient dead | 0 cases | … | … |  | … | … |  | … | … |  |
|  | 1-10 cases | … | … |  | 1.75 | 0.69 - 4.72 |  | … | … |  |
|  | 11+ cases | … | … |  | 8.83** | 1.95 - 46.29 |  | … | … |  |
| Fill out a death certificate | 0 cases | … | … |  | … | … |  | … | … |  |
|  | 1-10 cases | … | … |  | … | … |  | 2.0 | 0.87 - 4.88 |  |
|  | 11+ cases | … | … |  | … | … |  | 24.41** | 3.15 - 523.95 |  |
| Talk to a family after death | 0 cases | … | … |  | … | … |  | … | … |  |
|  | 1-10 cases | 0.79 | 0.32 - 1.97 |  | … | … |  | … | … |  |
|  | 11+ cases | 5.82* | 1.12 - 38.61 |  | … | … |  | … | … |  |

Supplemental Table 1: Increased OR refers to more agreement. PHD - Physician Hastened Death. OR – Odds ratio. PGY – Post Graduate Year. 95% CI – 95% Confidence Intervals. Statistical significance calculated as described in methods with *** p value < 0.001, ** p value < 0.01, * p value < 0.05

**CONTINUED SUPPLEMENTAL Table 1: Logistic Regression Examining Exposure to Death and Dying on Residents’ Willingness to Participate in MAID**

|  |  | **Administer Lethal Injection (#3) (n=185)** | | | **Administer Lethal Injection (#4) (n=184)** | | | **Administer Lethal Injection (#5) (n=184)** | | |
| --- | --- | --- | --- | --- | --- | --- | --- | --- | --- | --- |
| Variable | Category | OR | 95% CI |  | OR | 95% CI |  | OR | 95% CI |  |
| Age (in years) |  | 1.05 | 0.96 - 1.13 |  | 1.06 | 0.98 - 1.15 |  | 1.06 | 0.98 - 1.15 |  |
| PGY | 1 | … | … |  | … | … |  | … | … |  |
|  | 2 | 0.48 | 0.18 - 1.15 |  | 0.34* | 0.12 - 0.87 |  | 0.52 | 0.19 - 1.31 |  |
| Sex | Male | … | … |  | … | … |  | … | … |  |
|  | Female | 0.53 | 0.23 - 1.21 |  | 0.60 | 0.27 - 1.40 |  | 0.50 | 0.21 - 1.18 |  |
| School | Quebec | … | … |  | … | … |  | … | … |  |
|  | Ontario | 0.69 | 0.27 - 1.72 |  | 0.62 | 0.25 - 1.51 |  | 0.75 | 0.28 - 1.93 |  |
|  | Prairie | 0.50 | 0.13 - 1.75 |  | 0.53 | 0.15 - 1.67 |  | 0.50 | 0.14 - 1.63 |  |
| Religion | Not Religious | … | … |  | … | … |  | … | … |  |
|  | Other | 1.71 | 0.46 - 6.31 |  | 1.39 | 0.37 - 5.22 |  | 0.99 | 0.24 - 3.88 |  |
|  | Christian | 0.71 | 0.26 - 1.89 |  | 0.69 | 0.25 - 1.84 |  | 0.74 | 0.27 - 1.96 |  |
| Practice | Strictly | … | … |  | … | … |  | … | … |  |
|  | Not strictly | 7.45 | 1.15 - 153.45 |  | 5.22 | 0.86 - 101.20 |  | 8.93 | 1.32 - 191.41 |  |
|  | Not | 8.72 | 1.43 - 170.75 |  | 6.15 | 1.01 - 119.78 |  | 11.69* | 1.88 - 234.10 |  |
| Ethnicity | Other | … | … |  | … | … |  | … | … |  |
|  | Caucasian | 0.81 | 0.32 - 2.10 |  | 0.79 | 0.31 - 2.03 |  | 0.66 | 0.26 - 1.76 |  |
|  |  |  |  |  |  |  |  |  |  |  |
| Exposure to Death and Dying: | |  |  |  |  |  |  |  |  |  |
| Declare a patient dead | 0 cases | … | … |  | … | … |  | … | … |  |
|  | 1-10 cases | 1.50 | 0.54 - 4.50 |  | … | … |  | … | … |  |
|  | 11+ cases | 7.68* | 1.70 - 38.75 |  | … | … |  | … | … |  |
| Fill out a death certificate | 0 cases | … | … |  | … | … |  | … | … |  |
|  | 1-10 cases | … | … |  | 1.78 | 0.73 - 4.61 |  | … | … |  |
|  | 11+ cases | … | … |  | 9.46* | 1.49 - 69.29 |  | … | … |  |
| Talk to a family after death | 0 cases | … | … |  | … | … |  | … | … |  |
|  | 1-10 cases | … | … |  | … | … |  | 0.74 | 0.26 - 2.19 |  |
|  | 11+ cases | … | … |  | … | … |  | 6.82* | 1.35 - 39.53 |  |
